# A Systematic Review and Meta-Analysis of e-Mental Health Interventions to Treat Symptoms of Posttraumatic Stress

**DOI:** 10.2196/mental.5558

**Published:** 2017-05-17

**Authors:** Sara Simblett, Jennifer Birch, Faith Matcham, Lidia Yaguez, Robin Morris

**Affiliations:** ^1^ Institute of Psychiatry, Psychology and Neuroscience Department of Psychology King's College London London United Kingdom; ^2^ Springfield University Hospital South West London and St Georges Mental Health NHS Trust London United Kingdom; ^3^ Institute of Psychiatry, Psychology and Neuroscience Department of Psychological Medicine King's College London London United Kingdom; ^4^ King's College Hospital Clinical Neuropsychology Department King’s College Hospital NHS Foundation Trust London United Kingdom

**Keywords:** e-Mental Health, PTSD, psychological treatment, systematic review, meta-analysis

## Abstract

**Background:**

Posttraumatic stress disorder (PTSD) is a stress disorder characterized by unwanted intrusive re-experiencing of an acutely distressing, often life-threatening, event, combined with symptoms of hyperarousal, avoidance, as well as negative thoughts and feelings. Evidence-based psychological interventions have been developed to treat these symptoms and reduce distress, the majority of which were designed to be delivered face-to-face with trained therapists. However, new developments in the use of technology to supplement and extend health care have led to the creation of e-Mental Health interventions.

**Objective:**

Our aim was to assess the scope and efficacy of e-Mental Health interventions to treat symptoms of PTSD.

**Methods:**

The following databases were systematically searched to identify randomized controlled trials of e-Mental Health interventions to treat symptoms of PTSD as measured by standardized and validated scales: the Cochrane Library, MEDLINE, EMBASE, and PsycINFO (in March 2015 and repeated in November 2016).

**Results:**

A total of 39 studies were found during the systematic review, and 33 (N=3832) were eligible for meta-analysis. The results of the primary meta-analysis revealed a significant improvement in PTSD symptoms, in favor of the active intervention group (standardized mean difference=-0.35, 95% confidence interval -0.45 to -0.25, *P*<.001, I2=81%). Several sensitivity and subgroup analyses were performed suggesting that improvements in PTSD symptoms remained in favor of the active intervention group independent of the comparison condition, the type of cognitive behavioral therapy-based intervention, and the level of guidance provided.

**Conclusions:**

This review demonstrates an emerging evidence base supporting e-Mental Health to treat symptoms of PTSD.

## Introduction

Posttraumatic stress disorder (PTSD) is a stress disorder primarily characterized by four main symptom clusters: (1) unwanted intrusive re-experiencing of an acutely distressing, often life-threatening, event, (2) an ongoing state of hyperarousal, (3) an active avoidance of stimuli associated with an event that is perceived to be “traumatic”, and (4) negative thoughts or feelings that began or worsened after the trauma. Symptoms relating to PTSD are also found in people who may not have a formal diagnosis and can vary considerably in terms of severity. Other mental health problems such as poor anger control, drug and alcohol problems, and depression can develop alongside symptoms of PTSD, which can delay access to treatment and increase the burden of illness for individuals [1,2]. Research has shown that approximately half of those diagnosed with PTSD also suffer from major depressive disorder [3]. In most cases, depression typically improves during the course of treatment. However, some cases require specific treatment for depression prior to trauma-focused work. Disruptions to everyday functioning occurring within first 3 months following a “traumatic” event are classified by the *Diagnostic and Statistical Manual of Mental Disorders, 5th ed*. (DSM-5) [4] as an Acute Stress Reaction and PTSD thereafter. Prevalence rates calculated from data collected as part of the National Comorbidity Survey [5] estimate that 7.8% of people will experience PTSD at some point across their lifetime. This survey highlighted that some of the most commonly reported stressors were being directly involved in a life-threatening accident or critical illness; being involved in a fire, flood, or natural disaster; and being a witness to injury and threat to life or death of another person. Women were found to be at slightly higher risk than men (10.4 % vs 5.0%) [5]. Not all people who experience a trauma will develop PTSD, but factors that put people at greatest risk include prior trauma, family history of psychopathology, perceived life threat during the trauma, posttrauma social support, and peritraumatic dissociation [6].

Evidence-based psychological interventions have been developed to treat symptoms of PTSD, and the majority of these are designed to be delivered face-to-face using trained therapists. A recent systematic review and meta-analysis of psychological therapies for PTSD [7] described the evidence base for several types of psychological interventions. They separated trauma-focused cognitive behavioral therapy (TF-CBT) (eg [8]) from other forms of cognitive behavioral therapy techniques (non-TF-CBT). All the TF-CBT interventions involved exposure to aspects of the trauma such as revisiting the site where the traumatic event occurred with the aim of encouraging additional processing and updating the memory of the traumatic event, or “imaginal reliving” where individuals are supported to reimagine the trauma in sequence while vocalizing and reappraising the physical and cognitive reactions that occurred at the time of the event. The CBT interventions employed more general stress management techniques to help reduce anxiety (eg, relaxation). This paper also reviewed evidence for alternative psychological models including Eye Movement Desensitization and Reprocessing therapy (EMDR) [9,10] and psychodynamic approaches [11]. It was concluded that there were no differences in outcomes immediately posttreatment for TF-CBT, EMDR, and non-TF-CBT approaches, but that only TF-CBT and EMDR demonstrated sustained improvement between 1 and 4 months following treatment [7].

Access to evidence-based psychological interventions for PTSD remains a high priority in health care agendas, where screening and early interventions are recommended to prevent the development of more chronic presentations. However, mental health resources are often stretched and there is a need to think about extending access to such treatments. Information technology is becoming increasingly part of everyday life, and the possibility of seeking support and reputable information for mental health problems online is becoming more available. The Internet offers a means of accessing e-Mental Health resources for people in need of support, wherever they may be and at whatever time they require input. For people who are limited in their ability to access outpatient health services, be it due to funding, reduced physical mobility, lack of transport, or for fear of stigmatization, e-Mental Health services may be the most viable way for them to receive treatment in a timely and effective manner. Subclinical groups may benefit from evidence-based psychological approaches that prevent problems from worsening. There is an opportunity to increase accessibility of effective psychological support and empower people to take control and self-manage symptoms by embracing technological advances. This could either be via complete self-management or with some additional guidance.

There is already a good evidence base to support the effectiveness of e-Mental Health resources based on psychological models of therapy such as computerized CBT (cCBT) for treatment of depression and some anxiety disorders. Indeed, meta-analyses conclude that cCBT may be a very promising and efficacious treatment for depression within a diverse range of settings and clinical groups [12] as well as for panic disorder and phobia [13]. Research has shown that cCBT has the potential to be as effective as therapist delivered CBT [13-15], but that guidance yields better outcomes [12]. A number of previous systematic reviews and meta-analyses of e-Mental Health resources have included studies exploring treatments of PTSD (eg [16-20]) but only one recently published paper on e-Mental Health interventions for PTSD specifically [21]. This recent paper reviewed 20 randomized controlled trials of Web-based treatments for PTSD and concluded that CBT interventions significantly reduced symptoms compared to control conditions. They did not distinguish between CBT interventions that included an element of exposure as compared to more generic anxiety management tools, and they excluded studies that trialed interventions beyond the scope of CBT and expressive writing. A further systematic review of telepsychology has been published, which summarizes the evidence base to support interventions for PSTD provided remotely, for example, face-to-face therapy via videoconferencing technology [22]. However, this literature was considered beyond the scope of the current review, which aimed to focus on technology-supported interventions that enabled independent access to psychological treatment.

For the purpose of this review, e-Mental Health interventions are defined as psychological interventions delivered via the Internet through an interactive computer interface, including desktop and mobile devices. The aim was to evaluate the broader evidence base for e-Mental Health interventions (both Web-based and mobile-based) for treatment of symptoms associated with PTSD, specifically investigating the role of exposure exercises. In line with the latest review on face-to-face therapies for PTSD, the authors have retained the distinction made between TF-CBT and non-TF-CBT. EMDR and psychodynamic approaches were beyond the scope of this review as they have not yet been translated into e-Mental Health formats. The choice was to include a broad range of clinical and subclinical groups, including people treated within a physical health setting and without a formal diagnosis of PTSD, to assess the possible scope of such interventions to aid self-management.

## Methods

### Search Strategy

The following databases were searched in March 2015 using the keywords and phrases detailed in Table 1 along with equivalent Medical Subject Headings terms: Cochrane Library, MEDLINE, EMBASE, and PsycINFO. These searches were repeated in November 2016. Systematic reviews and meta-analyses published in the areas of technology-assisted self-help [19], computer-aided psychotherapy [17], and Internet or media-delivered CBT or other psychotherapies [16,18,20,21] for anxiety disorders and PTSD were hand searched to find studies. An additional systematic review on online interventions for cancer [23] was also hand searched. Trial registries and reference lists were searched for additional studies reporting analyzed results.

**Table 1:** Search terms for systematic review, combining Line 1 AND Line 2.

Search lines	Search terms	Filtered by
Line 1	PTSD OR (posttrauma* OR post-trauma*) AND (stress OR disorder*)	Title/Abstract
Line 2	(internet* OR web* OR tele* OR online OR “on-line” OR computer* OR mobile*) AND (“self-help” OR (self AND help) OR tool* OR resource* OR manual* OR package OR program* OR therap* OR intervention* OR application* OR technolog* OR device*) OR cCBT OR iCBT OR i-therapy OR itherapy OR e-therapy OR etherapy OR (virtual AND reality) OR avatar*	Title/Abstract

### Eligibility Criteria

The titles and abstracts for all identified papers were screened against the following inclusion criteria: (1) randomized controlled trial (RCT), (2) psychological therapy administered via a Web-based or mobile platform designed to treat symptoms of PTSD, (3) adults (aged 18 or over), (4) experience of a possible single-event trauma, and (5) assessed and reported symptoms of PTSD with a validated measure immediately after intervention. There was no restriction in terms of severity of PTSD symptoms or type of traumatic life event, and RCTs were included if they compared a waitlist, treatment as usual, or an active intervention. Interventions where participants received supplementary guidance from a therapist or other technical assistant were included and considered separately. Reasons for the exclusion of studies included technology that was not Web-based or mobile-based; study designs that were not RCTs including experimental manipulations, duplications, or interim or additional analysis; no measure of PTSD; an evaluation of complex intervention that included a Web-based intervention but did not exclusively test this; unpublished and unable to access results through personal communication; narrative literature reviews; and published protocols with no results.

### Study Selection

Two of the authors (SS and JB) read titles and abstracts of all potential papers independently and selected relevant articles for further review. Possible RCTs were read in full to determine if the trial met the inclusion criteria. This process was repeated after the second systematic search in November 2016 for articles published since March 2015.

### Data Extraction and Management

Literature searches were completed using reference manager software (Endnote X6 and Mendeley). Data were extracted from each paper on the (1) characteristics of each sample, including the population and demographic information such as the mean age or age range in years and the percentage of female participants, (2) characteristics of the treatment and control conditions, including the therapeutic model (for active conditions) and treatment duration, and (3) baseline and posttreatment scores on validated measures of PTSD. Authors were contacted directly for missing information. If the authors could not be contacted or did not respond, their papers were removed from the meta-analysis and included only in a narrative synthesis.

### Risk of Bias

All studies were subject to a structured quality assessment to investigate risk of bias using the Effective Public Health Practice Project (EPHPP) Quality Assessment Tool for Quantitative Studies [24]. This considered (1) selection bias, (2) study design, (3) confounders, (4) blinding, (5) data collection methods, and (6) withdrawals and dropouts. This is a standardized assessment tool that provides detailed guidance to aid classification of studies in terms of design quality. Additional areas of intervention integrity and appropriateness of the analysis are suggested as extra quality indicators but are not included in the total score on the measure. This information was collected and reported separately.

### Outcomes

The primary outcome of interest was severity of PTSD, measured continuously using a validated self-report or clinician-rated measures. This was qualified by the mean score value (M) and standard deviation (SD) on these measures.

### Characterization of the Interventions

#### Comparison Conditions

RCTs were subdivided and considered separately if they compared an active treatment condition to another active treatment condition or to a waitlist or treatment as usual (TAU) condition.

#### Model of Therapy

Studies were also considered separately depending on the therapeutic approach taken as part of active treatment condition. They were grouped as follows:

1. TF-CBT: treatment interventions that specifically involved exposure to aspects of the trauma and support to reappraise the physical and cognitive reactions that occurred at the time of the event.
2. Non-TF-CBT: treatment interventions based on the principles of CBT that employed more general stress management techniques to help reduce anxiety (eg, relaxation).
3. Other e-Mental Health interventions for treatment of PTSD: this category was included to capture treatment interventions that were not strictly designed based on CBT-based principles.

#### Guidance

Studies were further characterized by the level of guidance provided during this intervention and grouped as follows:

1. Individual tailored therapeutic feedback: feedback provided by a trained facilitator that related directly to the content of the therapeutic intervention.
2. Individual technical nontherapeutic support: support to facilitate technical functioning of the website or device that did not relate directly to the content of the therapeutic intervention.
3. Online discussion forum: a message board where participants could post messages and receive tailored feedback from a trained facilitator or peers.
4. Automated feedback only: feedback that was not individually tailored and was generated automatically in response to the completion of a task, for example.
5. Group feedback via videoconferencing: immediate feedback via videoconferencing provided by a trained facilitator or peers.
6. No guidance: no additional feedback or support in any form reported.

### Data Analysis

To assess the impact of intervention on total PTSD scores, standardized mean differences (SMDs) were calculated using postintervention mean and standard deviation values, with 95% confidence intervals (CI), as the primary outcome measures. Missing standard deviation values were calculated from the confidence intervals, standard errors, and sample sizes reported in the papers where possible and where this was not possible, additional information was requested from authors. In some cases, papers reported the median and interquartile ranges instead of the mean and standard deviation values and so this extra information was sought from authors. These data were pooled in a random-effects meta-analysis using Review Manager 5.0. The I^2^ statistic was calculated and used to assess heterogeneity of the studies included in each of the analyses. The following thresholds were followed: 0%-40% for unimportant heterogeneity, 30%-60% for moderate heterogeneity, 50%-90% for substantial heterogeneity, and 75%-100% for considerable heterogeneity [25].

Planned sensitivity analyses were conducted to test the robustness of the results. These included the exclusion of studies with a high risk of bias (defined as studies categorized as weak on the EPHPP tool) and where PTSD was not the primary outcome. Further planned subgroup analyses included the comparison condition (active comparison interventions vs waitlist or TAU control conditions), the model of therapy followed in the treatment condition (TF-CBT vs non-TF-CBT vs other models of therapy), and the type of guidance received during the treatment intervention (categorized as described in the section on guidance).

## Results

### Search Results

**Figure 1:**
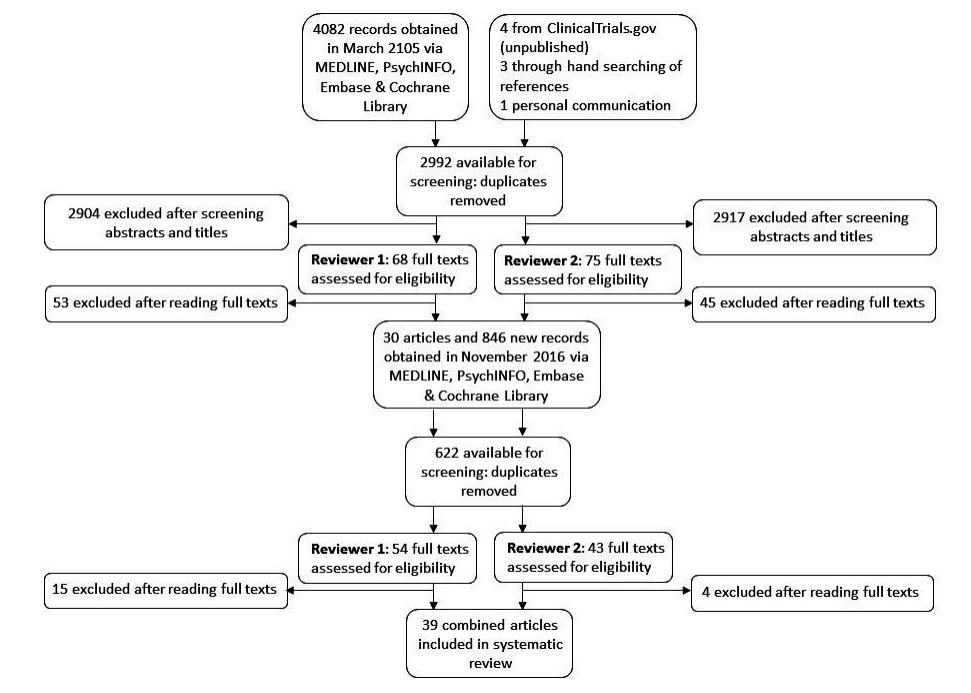
Flowchart of papers obtained and screened in the systematic search.

The search strategy conducted in March 2015 returned 2984 papers after duplicates were removed; 7 extra sources of information were obtained through hand searching or personal communication. A consensus was reached between two authors (SS and JB) that 30 RCTs met the inclusion criteria for the review. A further 9 papers were found following a repeat of this procedure in November 2016. Figure 1 shows a flowchart demonstrating this process for study selection of the final 39 papers included in the systematic review.

### Description of the Studies

A description of key information characterizing the studies included in this review is provided in Multimedia Appendix 1 (separated into studies that compared to an active treatment condition) and Table 2 (studies that compared to a waitlist control).

#### Sample Sizes

The number of participants included in the RCTs ranged from 25 [45] to 1292 [56]. We found 14 other studies that included sample sizes ≥100: Beyer [27], N=163; Brief [29], N=600; Carpenter et al [30], N=132; Cieslak et al [32], N=168; Hirai et al [37], N=133; Hobfoll et al [38], N=303; Kersting et al [41], N=228; Knaevelsrud et al [43], N=159; Lange et al [46], N=101; Marsac et al [49], N=100; Mouthaan et al [51], N=300; Schoorl et al [57], N=102; Spence [59], N=125; and Wang et al [62], N=183.

#### Study Country

The countries where the research studies were carried out included the US (n=19), the Netherlands (n=5), Australia (n=4), Sweden (n=3), Germany (n=3), Switzerland (n=2), Canada (n=1), Poland (n=1), and China (n=1).

#### Sample Characteristics

Seven studies included a sample who met full *Diagnostic and Statistical Manual of Mental Disorders*-IV (DSM-IV) diagnostic criteria for PTSD [28,39,42,43,57,58,62]. The majority of remaining papers reported on either university students [27,36,37,45], military service members and veterans [35,38,44,47,54], health and human service professionals [32], patients [26,30,51,53,55,63,64] or parents of patients [31,33,49] in medical settings, or other members of the wider community [40,41,46,48,50,52,61] self-reporting experience of a potentially traumatic event, receiving treatment from trauma-related services and or scoring high on a measure of PTSD, complicated grief, depression, or psychological distress. Two further studies included veterans self-reporting problems readjusting back into civilian life [56] or alcohol misuse [29]. Multimedia Appendix 1 provides further details of participant characteristics for each study.

#### Trauma Characteristics

Some studies focused on specific potentially traumatic events including bereavement [34,48,61] or loss of a child during pregnancy [40,41], physical injury or diagnosis of a chronic health condition such as cancer to oneself or a close other [26,30,31,33,49,51,53,63,64], organ transplant [55], complicated childbirth [52], as well as exposure to combat [29,35,38,44,47,54,56], sexual trauma [28], or natural disasters [60]. However, others did not specify the type of trauma experienced and included people with a multitude of difference experiences that could be potentially traumatic [36,37,39,42,43, 45,46,50,57-59,62]. Multimedia Appendix 1 provides further details of trauma characteristics, including time posttrauma.

**Table 2:** Quality assessment of RCTs of e-Mental Health interventions for treatment of symptoms of PTSD^a^.

Study author (year) [ref]	Selection bias	Study design	Confounders	Blinding	Data collection methods	Withdrawals and dropouts	Global rating for design methods (A-F)
Beatty (2016) [26]	•	•••	•••	••	•••	•••	••
Beyer (unpublished) [27]	••	•••	•••	••	•••	•••	•••
Bomyea (2015) [28]	••	•••	•••	•••	•••	••	•••
Brief (2013) [29]	•	•••	•••	••	•••	•	•
Carpenter (2014) [30]	•	•••	•••	•	•••	•••	•
Cernvall (2015) [31]	••	•••	•••	•	•••	••	•
Cieslak (2016) [32]	•	•••	•••	••	•••	•	•
Cox (2010) [33]	••	•••	•••	•	•••	••	••
Eisma (2015) [34]	•	•••	•••	••	•••	••	••
Engel (2015) [35]	•	•••	•••	••	•••	•••	••
Hirai (2005) [36]	••	•••	•••	••	•••	•••	•••
Hirai (2012) [37]	••	•••	•••	••	•••	••	•••
Hobfoll (2015) [38]	•	•••	•••	••	•••	•••	••
Ivarsson (2014) [39]	•	•••	•••	••	•••	•••	••
Kersting (2011) [40]	•	•••	•••	••	•••	••	••
Kersting (2013) [41]	••	•••	•	••	•••	•••	••
Knaevelsrud (2007) [42]	•	•••	•••	•	•••	•••	•
Knaevelsrud (2015) [43]	•	•••	•••	••	•••	•	•
Krupnick (unpublished) [44]	••	•••	•	••	•••	•	•
Lange (2001) [45]	••	•••	•	••	•••	•••	••
Lange (2003) [46]	•	•••	••	••	•••	•	•
Litz (2007) [47]	•	•••	•••	••	•••	••	••
Litz (2014) [48]	••	•••	•••	••	•••	•••	•••
Marsac (2013) [49]	••	•••	••	••	••	••	••
Miner (2016) [50]	•	•••	•••	••	•••	•••	••
Mouthaan (2013) [51]	•	•••	•••	••	•••	•	•
Nieminen (2016) [52]	•	•••	•••	••	•••	•••	••
Owen (2005) [53]	••	•••	•••	•	•••	•••	••
Possemato (2010) [54]	•	•••	•••	••	•••	•••	••
Possemato (2011) [55]	••	•••	•••	••	•••	•••	•••
Sayer (2015) [56]	•	•••	•••	••	•••	•••	••
Schoorl (2013) [57]	••	•••	•••	•••	•••	••	•••
Spence (2011) [58]	•	•••	•••	•	•••	•••	•
Spence (2014) [59]	••	•••	•••	•	•••	•••	••
Steinmetz (2012) [60]	••	•••	••	•	•••	•••	••
Wagner (2006) [61]	•	•••	•••	••	•••	•••	••
Wang (2013) [62]	•	•••	•••	••	••	••	••
Winzelberg (2003) [63]	•	•••	•••	••	•••	•••	••
Zernicke (2014) [64]	•	•••	•••	••	•••	•••	••

^a^•Weak; ••Moderate; •••Strong.

### Intervention Characteristics

#### Comparison Condition

Of the 39 RCTs included, 21 compared the e-Mental Health intervention with a waitlist control condition [29-31,33, 36,38,40-43,45,46,48,50,52,53,58,61-64]. See Multimedia Appendix 1 for further details on these studies.

The remaining studies compared to another active intervention, either instead of or as an additional arm to a waitlist control condition. Alternative active interventions included Web-based time management [27,54], Web-based factual writing [37,55,56], Web-based psychoeducation with [47] or without [26,32,60] supportive counseling, Web-based behavioral activation [34], Web-based weekly support, not specific to the traumatic event [39], an alternative computerized working memory capacity task [28], Web-based attention training [28,57], and treatment as usual within a clinical service [35,44,49,51,60].

One final study directly compared a version of a Web-based CBT intervention with and without exposure to the traumatic event [59]. Further details for all studies with an active comparison condition can be found in Multimedia Appendix 1.

#### Model of Therapy

The e-Mental Health interventions included in the meta-analysis incorporated a variety of therapeutic models. The interventions were grouped by similarities in theoretical methodologies as also described in Table 2 and Multimedia Appendix 1.

##### Trauma-Focused Cognitive Behavioral Therapy

We categorized 14 studies as TF-CBT [32,34,39-46, 52,58,59,61]. All of these interventions included exposure—be it indirect via imagery, written descriptions, or facilitated in vivo exercises—to the traumatic event and support to reappraise reactions. They also included additional CBT techniques such as psychoeducation about PTSD, self-monitoring of symptoms, behavioral activation, problem solving, goal setting, coping skills training (eg, relaxation), and cognitive restructuring. Half of these studies trialed the same Web-based intervention program: “Interapy” [40-43,45,46,61]. This Web-based intervention comprised three treatment phases: (1) self-confrontation where participants were required to write about the trauma focusing on the sensory perceptions in the present tense and in the first person, (2) cognitive reappraisal where participants were instructed to write a supportive and encouraging letter to a hypothetical friend who has been through a similar trauma with guidance on challenging unhelpful thinking and behavioral patterns, and (3) social sharing where, again, participants were asked to write a letter to another person but this time focusing on outlining difficult memories and reflecting on how they will cope with difficulties in the future.

##### Non‒Trauma-Focused Cognitive Behavioral Therapy

We grouped 16 studies as non-TF-CBT approaches that included CBT techniques, as detailed above, but did not include an element of exposure to the traumatic event and support to reappraise reactions [26,29-31,33,35,36,38,47-51,53,60,62]. Some incorporated other psychotherapeutic approaches, for example, motivational interviewing [29] or parenting guidance [33,49], in addition to CBT techniques. Others included additional elements of expressive writing, sometimes about a stressful event or trauma, but did not state that they supported participants to cognitively reappraise their reactions or provide any further therapeutic instruction focused on the trauma [30,31,36,47,51].

##### Other e-Mental Health Interventions

Nine studies trialed other types of e-Mental Health interventions including attentional bias modification [57], a working memory capacity task [28], expressive writing based on models of emotional disclosure without any additional CBT techniques [27,37,54-56], semistructured peer support only [63], and Mindfulness-Based Stress Reduction [64].

#### Guidance

In addition to this, interventions were further categorized into those that offered additional guidance. Some studies employed a combination of strategies to support completion. One study directly compared a Web-based intervention condition with and without guidance [27]. This study also compared two methods of providing guidance: delayed and immediate individual tailored feedback. The remaining studies were grouped as described in Table 2.

##### Individual Tailored Feedback

In addition to the study carried out by Beyer [27], a further 17 studies provided participants completing e-Mental Health interventions with individual tailored feedback [29,31,34,39-48, 52,58,59,61]. Seven of these studies stated that feedback was provided by either a licensed clinician (clinical psychologist, psychotherapist, psychiatrist) or clinician in training as a clinical psychologist or occupational therapist [31,34,39,42,43,58,61]. A further five studies reported that feedback was provided from graduates of clinical psychology courses who, in some cases, received additional training and clinical supervision but whose practicing status was not specified [45,46,48,59] or a nonclinical person such as a doctoral student who also received additional training and supervision from a licensed clinical psychologist [27]. The six remaining studies did not provide any details about the clinical experience of the person providing feedback [29,40,41,44,47,52]. Most of the individual tailored feedback was provided at a delay after the participants had completed a Web- or mobile-based module. However, one study in addition to the Beyer [27] study provided immediate individual tailored feedback through an instant messenger service [58] and another provided an initial session via telephone with a therapist at the start of the intervention [48].

##### Individual Technical Support

Two studies included intervention conditions where participants received individualized technical support but no tailored therapeutic guidance was offered [32,62].

##### Online Discussion Forum

Six studies included an online discussion forum where participants could interact with a licensed clinical psychologist or other mental health professional [58,63] or trained peer coach [38] or access self-guided peer support [30,51,53]. One of these studies provided additional automated guidance in the form of video-based vignettes of clinicians and cancer survivors [30], and another provided contact details for technical support [51].

##### Automated Feedback Only

One study provided only automated feedback on participants’ performance on a test of mastery [36].

##### Group Feedback Via Video-Conferencing

One study provided immediate feedback from both a behavioral medicine specialist and a group of peers completing the intervention live via video-conferencing technology [64].

##### No Guidance

A total of 12 studies reported on e-Mental Health interventions where participants were given no additional guidance [26,28,33,35,37,49,50,54-57,60].

### Measurement Tools

Tables 3 and 4 detail the outcomes of the studies included in this review for each of the standardized measures of PTSD employed.

The most commonly employed outcome measure of PTSD-related symptoms was the Impact of Events Scale (including the original IES, revised IES-R, and Dutch IES-D versions), which was administered in 16 studies [27,30,33,36,37,39-42,45,46,51-53,59,61]. The second most common measure was the PTSD Checklist (including the brief PCL-5, military PCL-M, and civilian PCL-C versions), which was administered in 13 studies [29,31,35,38,44,48-50, 54-56,58,63]. Other measures included the Posttraumatic Diagnostic Scale [39,43,62]; the Posttraumatic Stress Scale-Self Report version [26,34,47,59,63]; the Clinician Administered PTSD Scale [28,57]; the Secondary Traumatic Stress Scale [32]; the Self-Rating Inventory for Posttraumatic Stress Disorder [57]; the Modified PTSD Symptom Scale [60]; the Traumatic Event Scale [52]; and the Calgary Symptoms of Stress Inventory [64]. Four of the studies administered two measures of PTSD symptoms [36,39,52,57,59,63], while the remaining majority of studies administered only one.

### Studies Included in the Meta-Analysis

Six papers did not include sufficient information to be included in the meta-analysis (including a total score on the measure of PTSD), and their authors did not respond to our request for further information. Therefore, 33 papers were included in the final meta-analysis. Where studies included multiple condition groups (≥3), these were included in the analyses as separate comparisons. The sample size was adjusted in these analyses to account for multiple comparisons (eg, the total sample size was halved if a comparison of the same active intervention was made to two independent control condition groups). In total, 5405 participants were included in the studies. Data from 3832 participants contributed to the meta-analyses.

### Risk of Bias

Results of the quality assessment for risk of bias performed on the studies included in this systematic review can be found in Table 2. Seven of the studies (17.9%) demonstrated the highest possible overall rating for quality of the design [27,28,36,37,48,54,57]. Marks were most frequently lost for methods of sampling bias, with a large proportion of studies recruiting via a relatively unsystematic and opportunistic method [26,29,30,32,34,35,38-40,42,43,46,47,50-52, 55,56,58,61-64]. Strict criteria for blinding of participants and assessors were met in only two studies [27,57]. For clarification, when reviewing the quality of blinding across the RCTs, it was not always possible to identify with certainty that participants or outcome assessors were blind to the conditions of the study if it was not explicitly stated by the authors. Therefore, it was agreed that cases of ambiguity were given the benefit of the doubt and rated for blinding as moderate rather than weak according to the quality of evidence tool. However, this means that studies that did clearly state that conditions were not blinded may have been awarded a lower rating than those that omitted this information. Retention of participants in the studies was relatively good, with 23 of the studies (59.0%) retaining over 80% of participants throughout the intervention and through to postcompletion assessment [26,27,30,35,36,38,39,41,42,45,48, 50,52-56,58-61,63,64]. Only six studies (15.4%) failed to control for at least 90% of potential confounders in the analysis [41,44-46,49,60] [49,62] employed only standardized measured to assess outcomes, highlighting a further relative strength in the design of studies conducted within this area.

### Treatment Effects

#### Impact of the Interventions on Posttraumatic Stress Disorder

A meta-analysis on the between-group difference for end-point scores on measures of PTSD demonstrated a significant effect in favor of the treatment group (SMD -0.35, 95% CI -0.45 to -0.25, *P*<.001). However, there was considerable heterogeneity between the study comparisons made (I^2^=81%).

Of the six studies that were not included in the meta-analysis, four studies found a significant difference on all subscales representing dimensional symptoms of PTSD, including intrusions, avoidance, and hyperarousal, in favor of the treatment intervention [42,45,46,61]. Sayer et al [56] found a significant between-group difference on total PTSD scores favoring an active intervention of expressive writing compared to a no writing control condition. Another study reported no significant between-group difference on a clinician-rated measure of PTSD [54].

#### Sensitivity Analyses

The planned sensitivity analyses (Table 3) demonstrated that the outcomes for PTSD were relatively stable. The exclusion of studies with a high risk of bias increased the effect size slightly in comparison to the primary analysis, whereas the excluded studies without PTSD as a primary outcome slightly increased the effect size compared to the primary analysis. For all analyses, the heterogeneity of the studies remained considerably high.

**Table 3:** Impact of Web-based interventions on PTSD: sensitivity analyses.

Analysis	Comparisons, n	Participants, n	SMD^a^ (95% CI)	*P*	I^2^ statistic, %
Primary analysis	38	3832	-0.35 (-0.45 to -0.25)	<.001	81
Excluding studies with high risk of bias	30	2340	-0.36 (-0.50 to -0.21)	<.001	81
Excluding studies without PTSD as a primary outcome	35	3551	-0.34 (-0.44 to -0.24)	<.001	80

^a^SMD >0 favors control, and SMD <0 favors active intervention.

#### Subgroup Analyses

**Figure 2:**
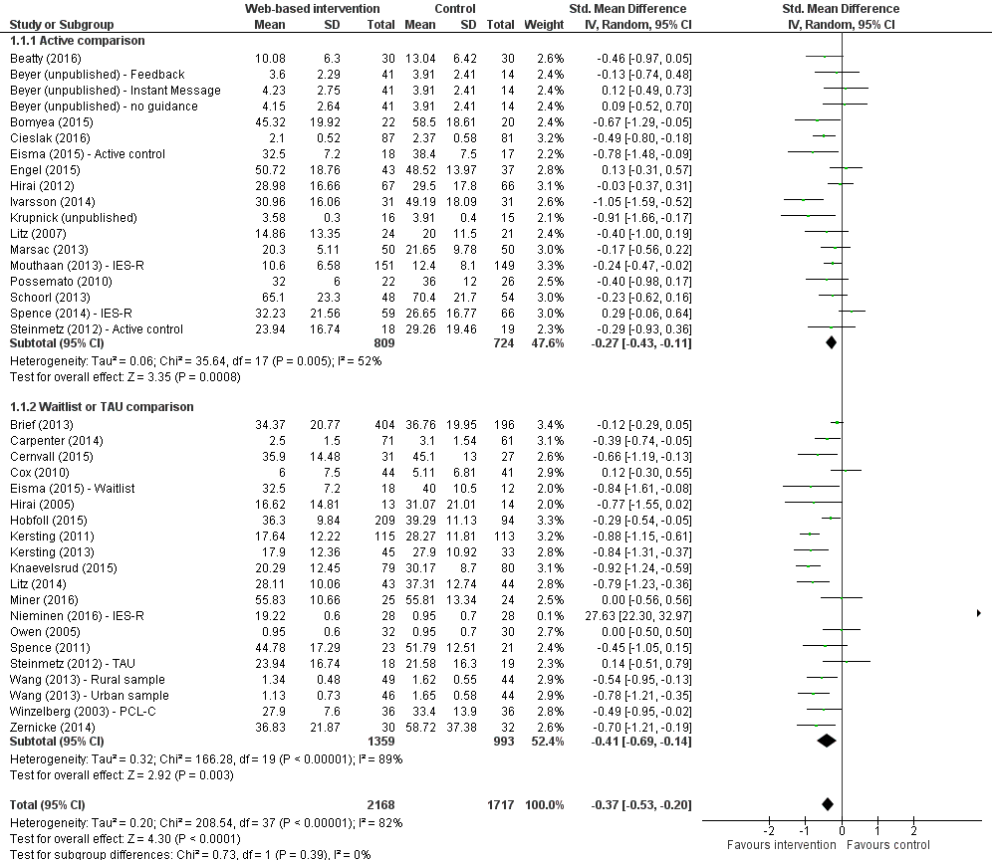
Impact of e-Mental Health interventions on PTSD, divided by comparison group.

The first planned subgroup analysis involved separating studies into those that compared an active intervention to another active comparison condition and those that compared to either a waitlist control or treatment as usual. Figure 2 shows the impact of e-Mental Health interventions on end-point outcomes for PTSD, split by comparison group. A significant effect in favor of the treatment group remained for both subgroups of studies that compared to another active intervention condition (SMD -0.27, 95% CI -0.43 to -0.11, *P*<.001, I^2^=52%) and either a waitlist or TAU control condition (SMD -0.41, 95% CI -0.69 to -0.14, *P*<.001, I^2^=89%), with effect sizes slightly greater for the latter subgroup. However, the heterogeneity of the studies was improved for studies compared to another active intervention condition. Table 4 compares this subgroup analysis to other planned subgroup analyses including the model of therapy and the type of guidance received.

**Table 4:** Impact of Web-based interventions on PTSD: subgroup analyses.

Analysis	Subgroups		Comparisons, n	Participants, n	SMD^a^ (95% CI)	*P*	I^2^ statistic, %
Primary analysis			38	3832	-0.35 (-0.45 to -0.25)	<.001	81
**Comparison group**							
	Active		18	1533	-0.27 (-0.43 to -0.11)	<.001	52
	Waitlist or TAU		20	2352	-0.41 (-0.69 to -0.14)	<.001	89
**Model of therapy**							
	**TF-CBT**		11	997	-0.34 (-0.48 to -0.21)	<.001	92
		Interapy	3	465	-10.24 (-12.32 to -8.15)	<.001	0
		Other	8	532	-0.30 (-0.44 to -0.16)	<.001	77
	Non-TF-CBT		18	2227	-0.36 (-0.50 to -0.22)	<.001	62
	Expressive writing		5	346	-0.04 (-0.88 to 0.79)	.92	0
	Attention bias modification		1	102	—	—	—
	Working memory capacity task		1	42	—	—	—
	Semistructured peer support		1	72	—	—	—
	Mindfulness-based stress reduction		1	62	—	—	—
**Guidance**							
	Individual tailored feedback		16	1743	-0.52 (-0.76 to -0.28)	<.001	90
	Individual technical support		2	261	-0.27 (-0.40 to -0.14)	<.001	0
	Online discussion forum		6	913	-0.26 (-0.53 to 0.01)	.06	72
	Automated feedback only		1	27	—	—	—
	Live group feedback		1	62	—	—	—
	No guidance		13	927	-0.50 (-0.76 to -0.24)	<.001	13

^a^SMD > 0 favors control, and SMD < 0 favors active intervention.

The additional subgroup analyses showed that studies grouped together as following TF-CBT protocols were not only significantly effective compared to controls but were as effective as studies grouped together as following non-TF-CBT protocols. The heterogeneity of TF-CBT was high, falling within the substantial range. A subgroup analysis grouping together only those studies that trialed the intervention package, Interapy, demonstrated an unimportant level of heterogeneity, while retaining a significant between-group difference. The large variation in heterogeneity can be accounted for the other studies grouped together as TF-CBT interventions. Web-based expressive writing interventions did not significantly differ from their control conditions but demonstrated an unimportant level of heterogeneity suggesting that these interventions were more comparable. In terms of the single studies that could not be included in the meta-analysis, the attention bias modification training was reported to be equally effective at reducing symptoms of PTSD and an active control condition [57]. The working memory capacity training was found to be significantly effective at reducing the re-experiencing of symptoms over and above an active control condition, but no significant interaction effects were found on other dimensions of PTSD symptoms including avoidance and arousal [28]. The Web-based mindfulness-based stress reduction intervention resulted in significant improvements in PTSD symptoms relative to a waitlist control condition [64].

For the subgroup analysis of guidance, groups of studies demonstrated a significant between-group difference regardless of whether guidance was provided in the form of individual tailored feedback during the therapeutic intervention, as individual technical support only or if no guidance was provided at all. The heterogeneity of studies included in the subgroup analysis was the lowest for no guidance group. Both the individual tailored feedback group and the individual technical support group demonstrated at least a substantial degree of heterogeneity. The only subgroup that did not show a significant between-group difference included the studies that had only an online discussion forum. A meta-analysis could not be carried out on the two remaining groups: automated feedback only and live group feedback, given that only a single study included this method in each case. However, in terms of the results described in these papers, Hirai and Clum [36] found that participants who received an 8-week Web-based program for traumatic event-related consequences with only automated feedback reported a significant decrease in avoidance behavior and frequency of intrusive symptoms as compared to participants on a waiting list. As previously reported Zernicke [64], who used live group feedback via videoconferencing technology, demonstrated significant improvements in PTSD symptoms relative to a waitlist control condition.

## Discussion

### Principal Findings

This systematic review and meta-analysis summarizes the results of RCTs designed to assess the impact of e-Mental Health interventions on symptoms of PTSD. There is some evidence to suggest that these e-Mental Health treatments are effective at reducing PTSD-related distress over and above a variety of control conditions. Results from the primary meta-analysis of endpoint data showed a medium and significant between-group difference in PTSD symptoms, in favor of the active intervention conditions. However, there was considerable heterogeneity between the studies included in this review, raising some concerns about how meaningful it is to make direct comparisons between the studies included in this meta-analysis. The results remained similar when removing studies that were found to present a high risk of bias, suggesting that factors other than those contributing the quality assessment scores may have accounted for the high level of heterogeneity between studies.

When dividing studies by the characteristics of the condition to which they were compared, the results became slightly clearer. A medium and significant between-group difference was found for studies compared to either a waitlist control or TAU condition, favoring the active intervention. While still favoring the active intervention, only a small significant between-group difference was found for studies compared to another active condition. Studies compared to an active intervention were much less heterogeneous, providing some further confidence to support the reliability of these estimated effects. This evidence suggests that not only is there some good evidence emerging to support the efficacy of e-Mental Health treatments for PTSD but that these interventions contain an active component that is stronger than other Web- or mobile-based activities including psychoeducation, behavioral activation, weekly non-specific support, and factual writing about a traumatic event.

In a further subgroup meta-analysis, we explored the impact of different models of therapy. A medium and significant between-group difference favoring the active intervention was found for both studies categorized as employing TF-CBT techniques and non-TF-CBT techniques. The main difference between these interventions was that the former required participants to take part in exercises that encouraged them to re-engage with the trauma and work on reappraising the cognitive and physical consequences of the event, while the latter included CBT techniques with a more general focus on anxiety and stress management. From this analysis, there appears to be little difference between the two groups, with both being as effective as the other at reducing symptoms of PTSD. However, it is still important to note that these two subgroups of studies still demonstrated a high degree of heterogeneity, suggesting some qualitative differences between studies included in the analysis. CBT is a broad term, encompassing a wide range of therapeutic techniques. In addition to this, some studies reported incorporating techniques from alternative models of therapy, such as motivational interviewing, alongside CBT techniques. It is possible that some of the heterogeneity between studies could be accounted for by differences in the therapeutic techniques employed. When comparing studies that used the same Web-based treatment program (Interapy), the results were much less heterogeneous and remained significant and in favor of the active intervention condition. However, the between-group difference reduced to a small effect. This result was limited by the exclusion of four additional studies that trialed the efficacy of the Interapy program, as total PTSD scores could not be gathered from all the authors.

In relation to the alternative models of therapy trialed in previous RCTs for treatment of PTSD, as yet, there is little evidence to support the use of Web-based attentional bias modification paradigms, and only some emerging evidence to support a working memory capacity paradigm and mindfulness-based stress reduction techniques from single studies alone. A meta-analysis of expressive writing, without any additional CBT techniques, showed a nonsignificant difference between active intervention and control groups. There is scope to explore some of these alternative therapy models in greater depth. Face-to-face mindfulness-based approaches have been gathering evidence for treatment of recurrent and chronic depression, anxiety, and stress [65]. However, there currently is a far greater body of evidence in favor of CBT-based techniques presented through e-Mental Health sources for treatment of PTSD.

For the final subgroup analysis, we explored the impact of the type of guidance or support that participants received while completing e-Mental Health interventions. Medium-sized, significant group-differences, favoring the active interventions were found regardless of whether participants received individual tailored feedback or no guidance. Studies that provided participants with merely technical support demonstrated a small significant between-group difference in favor of the active intervention. This suggests that while previous studies have indicated that guidance improves outcomes for e-Mental Health interventions [12], this was not the finding of a meta-analysis of studies trialing e-Mental Health interventions for treatment of PTSD. It is important to mention that the type of individual tailored feedback varied greatly between the studies, specifically in terms of the qualifications and experience of the person providing feedback, which sometimes was reported to vary even within single studies. The impact of this was not explicitly tested in the analysis conducted here and could be an avenue for future research.

The only subgroup that did not demonstrate a significant between-group difference consisted of studies that had facilitated support with the e-Mental Health intervention via an online discussion forum. This was the only group-based means of providing feedback included in the meta-analysis. Emotions such as shame, guilt, and fear of negative evaluation from others are commonly associated with PTSD [66-68] and can present barriers for open communication about the impact of trauma. Arguably, the added anonymity of Web-based or mobile resources may provide some advantage over therapist-delivered treatments. However, research is needed to test the empirical validity of this hypothesis, especially when using online discussion forums between groups of people. Interestingly, the study employing mindfulness-based stress reduction techniques via an online forum, which reported a significant between-group difference in favor of the active intervention, included live group interaction via video-conferencing technology. Further research would be required to establish whether this positive effect on reducing symptoms of PTSD was facilitated by the model of therapy or type of guidance employed.

### Strengths and Limitations

This review considered RCTs of e-Mental Health resources for treatment of PTSD-related symptoms, including TF-CBT, non-TF-CBT, and other psychological therapies. It encompassed research on trauma spanning different countries, including people with different experiences in terms of the characteristics of the trauma and severity of PTSD-related symptoms. Many of the studies demonstrated strengths in design, assessment, and analysis of results. The results are consistent with but also extend previous findings; this study reports a significant between-group difference when comparing active interventions to active control conditions as well as more passive waitlist or TAU control conditions [21]. This is also consistent with the previous meta-analysis in that guidance was not found to moderate treatment outcomes. However, there are a number of limitations to this review.

This review did not consider acceptability of the interventions. Quality assessment indicated that studies varied in terms of participant retention, and it may be worthwhile to perform an analysis of dropout rates in an addition to the outcomes already assessed in this review. As people may be given access to e-Mental Health resources in their own homes without direct supervision, it is paramount that the risks and potential adverse effects associated with completing these type of interventions are thoroughly investigated. Finally, there were variations between studies in terms of treatment adherence, that is, the level of engagement necessary to constitute an episode of treatment. Some studies required submission of a set number of essays by participants, whereas others were monitored simply by the frequency of logging in to the intervention. For future research, determining the optimal level of engagement for therapeutic benefit of online treatments needs to be investigated in order to establish model fidelity of interventions and permit consistent clinical application.

This review focused on the use of e-Mental Health resources for treatment of PTSD. Only one study reported an RCT of a mobile-based treatment for PTSD. With the development of new mobile-based treatments, it is likely that future reviews may be able to make comparisons between Web-based and mobile-based technologies. Also missing from the scientific literature at present are studies that compare the relative efficacy of Web- or mobile-based and face-to-face therapist-delivered interventions for treatment of PTSD. In addition, there are limited follow-up data to assess the long-term benefits of interventions trialed. The review highlighted a number of weaknesses in the design of past studies particularly in relation to sample methods, the most notable being the unequal gender balance seen in most studies to date.

### Conclusions

Research in this field is developing at a fast pace with creation of new technologies ever increasing. Future studies need to focus on maintaining a high quality of assessment of the efficacy and acceptability of these technologies in the face of these rapid developments. Assessment of side effects and risks should not be overlooked, despite the potential for interventions being more readily accessible. Replications of findings are needed to investigate the use of similar e-Mental Health interventions across diagnostics groups and health settings and could benefit from research to better understand which specific intervention packages or components work best and for whom. Related to this is the issue of gaining a better understanding of more practical factors influencing outcome such as an individual’s technological literacy, the ease of use given graphical interface designs, and the mode of delivery (eg, via computers or apps on mobile and tablet devices). There is scope for developing more user-friendly tools. Collaborations between software engineers and designers, who have the ability to build the technology; psychologists, who have the theoretical knowledge of evidence-based therapeutic interventions; and individuals trialing prototypes, who have a unique expertise in their implementation, will be very important for creating user-friendly and effective resources. There is far to go in terms of gathering the same level of evidence base as therapist-delivered approaches. However, the results presented in this systematic review take a small step forward in understanding how technology such as e-Mental Health resources may offer additional opportunities for increasing access to effective psychological support for people suffering from PTSD, to improve well-being.

## Appendix

Multimedia Appendix 1 Characteristics of studies.

## Abbreviations

CBT: cognitive behavioral therapy
cCBT: computerized cognitive behavioral therapy
CI: confidence interval
DSM: Diagnostic and Statistical Manual of Mental Disorders
EMDR: Eye Movement Desentitization Reprocessing Therapy
EPHPP: Effective Public Health Practice Project
IES: Impact of Events Scale
IES-D: Impact of Events Scale-Dutch version
IES-R: Impact of Events Scale-revised
Non-TF-CBT: non‒trauma-focused cognitive behavioral therapy
PCL-5: PTSD Checklist-5
PCL-C: PTSD Checklist-Civilian Version
PCL-M: PTSD Checklist-Military Version
PTSD: posttraumatic stress disorder
RCT: randomized controlled trial
SMD: standardized mean differences
TAU: treatment as usual
TF-CBT: trauma-focused cognitive behavioral therapy
