# Supplementary material for: A Systematic Review and Meta-Analysis of e-Mental Health Interventions to Treat Symptoms of Posttraumatic Stress
Source: JMIR Ment Health. 2017 May 17;4(2):e14. doi: 10.2196/mental.5558 (PMC5451639; doi:10.2196/mental.5558)
Supplement: Multimedia Appendix 1 [file mental_v4i2e14_app1.pdf]

Appendix 1. Characteristics of studies where interventions were compared to an active treatment.

Study: author (year)	Active Intervention (n)	Control (n)	Treatment duration (follow-up duration)	Population	Mean age (age range) in years	% female	Measure	Baseline scores
Beatty et al. (2016)	Web-based non-TF-CBT with no guidance (30)	Web-based psychoeducation with no guidance (30)	6 sessions (7 weeks)	Men and women with a diagnosis of cancer ≤6 months previously, receiving treatment with curative intent	Active: 51.6±10.1 Control: 53.9±9.5 (30-84)	95	PSS	Active: 13.1±10.5 Control: 11.2±7.9
Beyer (unpublished)	Web-based expressive writing (123): T1: no guidance (41) T2: with delayed tailored feedback (41) T3: with immediate tailored feedback (41)	Web-based time management writing with no guidance (41)	3 sessions of 40 minutes scheduled 7-10 days apart (6 weeks)	University students self-reporting experience of a traumatic event; time post trauma not specified	21.9±6.6 (17-61)	83.4	IES-R	Active: T1: 5.7±2.8, T2: 4.8±2.1, T3: 5.2±2.1, Control: 5.4±2.1
Bomyea et al. (2015)	Computerised working memory capacity task with no guidance (22)	Computerised working memory capacity task with low interference and no guidance (20)	8 sessions over four weeks (9 weeks)	Women meeting DSM-IV criteria for PTSD secondary to a sexual trauma; >3 months post trauma	Active: 29.8±10.7 Control: 26.0±10.6	100	CAPS	Active: 61.4±12.5 Control: 67.4±15.0
Brief et al. (2013)	Web-based non-TF-CBT and MI with tailored feedback (404)	Waitlist (196)	8 weekly sessions of 20 minutes (8 weeks)	Veterans self-reporting alcohol misuse; time post trauma not specified	Active: 32.0±7.8 Control: 32.1±7.7	13.7	PCL-5	Active: 41.5±19.5 Control: 39.4±18.7
Carpenter et al. (2014)	Web-based non-TF-CBT with automated guidance and an online discussion forum (71)	Waitlist (61)	10 weekly sessions; one chapter per week (10 weeks and 20 weeks)	Women diagnosed with breast cancer ≤18 months; currently stage 0, I, II or III scoring high on measures of psychological distress	50.9±9.9 (25-73)	100	IES-R	Total: 3.6±0.2
Cernvall et al. (2015)	Web-based non-TF-CBT with tailored feedback (31)	Waitlist (27)	10 weekly sessions in structured modules (10 weeks)	Parents of children diagnosed with cancer (4-12 weeks previously; mean=3.0±3.0) scoring high on a measure of PTSD	38±7.2	67 (38% both parents included)	PCL-C	Active: 51.5±9.4 Control: 46.6±10.7
Cieslak et al. (2016)	Web-based TF-CBT with technical support (87)	Web-based psychoeducation with technical support (81)	4 sessions over 4 weeks (1 month and 2 months)	Health and human service professional exposed indirectly to traumatic events at work; >1 year post trauma	37.5±10.4	78.0	STSS	Active: 2.3±0.6 Control: 2.4±0.7
Cox et al. (2010)	Web-based non-TF-CBT with additional parenting intervention with no guidance (44)	Waitlist (41)	Open access for 5 months (4-6 weeks and 6 months)	Parents/guardians of children (aged between 7 and 16 years) who had sustained an accidental or unintentional physical injury, including mild traumatic brain injury; time post trauma was within 72 hours	Active: 41.2±7.0 Control: 40.1±6.4	88	IES-R	Active: 10.7±10.5 Control: 9.5±9.5

Study: author (year)	Active Intervention (n)	Control (n)	Treatment duration (follow-up duration)	Population	Mean age (age range) in years	% female	Measure	Baseline scores
Eisma et al. (2015)	Web-based TF-CBT with tailored feedback (18)	Web-based behavioural activation with delayed therapist feedback (17)<br><br>Waitlist (12)	6 e-mail assignments completed over a 6-8 week period	Men and women who had experienced a death of a first-degree relative more than 6 months previously and reported elevated levels of complicated grief	45.7±12.9	91.5	PSS	Active: 37.3±5.2 Control: 37.7±9.0
Engel et al. (2015)	Web-based non-TF-CBT with no guidance (43)	TAU through primary care clinic (37)	3 sessions per week lasting 6 weeks, 15-30 minute sessions with 30 minute homework tasks (6 weeks, 12 weeks and 18 weeks)	Military service members and veterans screening positive for PTSD; time post trauma not specified	Active: 36.2±7.8 Control: 36.7±9.8	18.8	PCL-C	Active: 58.6±10.0 Control: 55.2±10.9
Hirai et al. (2005)	Web-based non-TF-CBT with automated feedback (13)	Waitlist (14)	8 weekly sessions (8 weeks)	University students self-reporting experience of a traumatic event accompanied by re-experiencing and avoidance symptoms of PTSD; time post trauma not specified	29.4±11.5	77.8	IES-R	Active: 41.8±12.1 Control: 46.9±20.3
Hirai et al (2012)	Web-based emotional expressive writing with no guidance (67)	Web-based factual writing with no guidance (66)	3 sessions on consecutive days (1 week – mid-intervention and 5 weeks)	Undergraduate students (identifying as Hispanic American or Mexican) self-reporting experience of a traumatic event; time post trauma not specified	23.9±5.4 (18-48)	84.6	IES-R	Active: 41.2±16.2 Control: 38.5±15.1
Hobfoll et al. (2015)	Web-based non-TF-CBT with an online discussion forum and peer chat support (209)	Waitlist (94)	Open access to 7 modules spread at least 2 days apart over 6 weeks (6 weeks and 12 weeks)	Veterans scoring in at least the mild (but not severe) range on measures of PTSD and depression; time post trauma not specified	Active: 34.2±7.6 Control: 34.7±8.9	18.5	PCL-M	Active: 40.0±11.2 Control: 37.5±11.5
Ivarsson et al. (2014)	Web-based TF-CBT with tailored feedback (31)	Web-based weekly non-specific support (31)	8 weekly sessions (8 weeks)	Community-based sample meeting DSM-IV criteria for PTSD; >3 months post trauma	46±11.7 (21-67)	82.3	IES-R<br><br>PDS	Active: 54.7±13.2 Control: 54.9±15.5<br><br>Active: 31.9±6.5 Control: 29.8±8.8
Kersting et al. (2011)	Web-based TF-CBT with tailored feedback (45)	Waitlist (33)	2 sessions per week lasting 5 weeks, 45 minutes each (5 weeks)	Women who had lost a child during pregnancy; mean time since loss was 15.4±27.4 months	34.3±5.3	100	IES	Active: 33.1±13.2 Control: 34.6±11.4
Kersting et al. (2013)	Web-based TF-CBT with tailored feedback (115)	Waitlist (113)	2 sessions per week lasting 5 weeks, 45 minutes each (5 weeks)	Parents who had lost a child during pregnancy; mean time since loss was 9.9±24.1 months	34.2 ± 5.2	92.1	IES-R	Active: 17.4±7 Control: 19.2±6.8

Study: author (year)	Active Intervention (n)	Control (n)	Treatment duration (follow-up duration)	Population	Mean age (age range) in years	% female	Measure	Baseline scores
Knaevelsrud et al. (2007)	Web-based TF-CBT with tailored feedback (49)	Waitlist (47)	2 sessions per week lasting 45 minutes, over 5 weeks; 10 sessions in total (5 weeks)	Community-based sample meeting DSM-IV criteria for PTSD; >3 months post trauma	Active: 34±11.5 Control: 36±9.6 (18-68)	90	IES-R	Intrusions: Active: 23.0±6.4 Control: 23.3±7.9 Avoidance Active: 19.9±9.8, Control: 19.0±10.0, Hyper arousal: Active: 22.1±6.5 Control: 19.1±9.5
Knaevelsrud et al. (2015)	Web-based TF-CBT with tailored feedback (79)	Waitlist (80)	2 sessions per week lasting 45 minutes, over 5 weeks; 10 sessions in total (5 weeks and 3 months)	Arabic-speaking adults meeting DSM-IV criteria for PTSD; >3 months post trauma	Active: 29.12±8.2 (18-56) Control: 27.2±6.5 (18-43)	91.2	PDS	Active: 30.4±8.2 Control: 30.7±8.1
Krupnick et al. (unpublished)	Web-based TF-CBT with tailored feedback (16)	Psychosocial treatments and antidepressant medication (15)	10 sessions (12 weeks and 24 weeks)	Veterans being treated in trauma services; time post trauma not specified	40 (27-64)	88.2	PCL-M	Active: 3.6±0.3 Control: 3.9±0.4
Lange et al. (2001)	Web-based TF-CBT with tailored feedback (69)	Waitlist (32)	2 sessions per week lasting 45 minutes, over 5 weeks; 10 sessions in total (6 weeks)	Community-based sample of visitors to a therapy website scoring high on measures of PTSD; time post trauma not specified	39.0±10.5 (19-71)	80	IES-D	IES-D Intrusions: Active: 20.2±7.5 Control: 19.9±8.2<br><br>IES-D Avoidance: Active: 14.1±8.0 Control: 16.8±8.5
Lange et al. (2003)	Web-based TF-CBT with tailored feedback (13)	Waitlist (12)	2 sessions per week lasting 45 minutes, over 5 weeks; 10 sessions in total (6 weeks)	University students self-reporting experience of a traumatic event >3 months previously	22±4.9 (18-37)	64	IES-D	IES-D Intrusions: Active: 17.5±6.5 Control: 13.6±7.0<br><br>IES-D Avoidance: Active: 12.5± 8.6 Control: 11.7± 8.6
Litz et al. (2007)	Web-based non-TF-CBT with initial face-to-face therapy session and subsequent tailored feedback via e-mail and telephone contact (24)	Web-based psycho-education and supportive counselling with delayed therapist feedback via e-mail and immediate contact via telephone (21)	7 weekly sessions (8 weeks post, 3 months follow-up, 6 months follow-up)	Veterans scoring high on measures of PTSD; time post trauma not specified	Active: 39.9±7.7 Control: 38.6±9.4	19	PSS	Active: 29.2±9.9 Control: 26.7±9.0

Study: author (year)	Active Intervention (n)	Control (n)	Treatment duration (follow-up duration)	Population	Mean age (age range) in years	% female	Measure	Baseline scores
Litz et al. (2014)	Web-based non-TF-CBT with an initial session with a therapist via telephone and subsequent tailored feedback via e-mail as required (43)	Waitlist (44)	18 sessions lasting 20 minutes each, 3 sessions weekly encouraged but participants were allowed to work at own pace (6 weeks)	Men and women recently bereaved (loss of a relative receiving treatment through Oncology service between 3 and 6 months) scoring high on a measure of prolonged grief disorder with functional impairment	55.4±10.3	68	PCL-C	Active: 39.7±12.0 Control: 38.3±11.3
Marsac et al. (2013)	Web-based non-TF parenting intervention with elements of CBT and no guidance (50)	Psychosocial care from a specialist trauma centre(50)	1 20-minute session with open access over 1 month (6 weeks)	Parents of children (aged 6-17 years) who had incurred an injury and received medical treatment at a paediatric trauma centre (≤60 days previous)	41.02±7.50 (23-59)	83	PSS	Active: 23.3±7.6 Control: 24.2±9.9
Miner et al. (2016)	Mobile-app non-TF-CBT with no guidance (25)	Waitlist (24)	Open access to mobile app over a 1 month period (1 month)	Men and women self-reporting experience of a trauma scoring high on a measure of PTSD; time post trauma not specified	45.7±13.9	81.6	PCL-C	Active: 63.0±11.3 Control: 59.3±11.3
Mouthaan et al. (2013)	Web-based non-TF-CBT with contact information for assistance provided and an online discussion forum (151)	TAU including psychosocial care from a special trauma centre and primary care provider (149)	1 30 minute session with open access over 1 month (1, 3, 6 and 12 months)	Men and women admitted to an acute trauma centre for a physical injury	43.8±15.9	40	IES-R	Active: 17.6±16.8 Control: 21.2±19.1
Nieminen et al. (2016)	Web-based TF-CBT with tailored feedback (28)	Waitlist (28)	8 modules presented one per week over 8 weeks (8 weeks)	Women in the postpartum period at least three months since delivery scoring high on a measure of PTSD; time post-delivery was within 6.5 years, mean=2.8 years	34.6±4.8	100	TES<br><br>IES-R	Active: 42.7±9.7 Control: 40.2±11.0<br><br>Active: 39.8±12.9 Control: 27.1±16.7
Owen et al. (2005)	Web-based non-TF-CBT with no guidance but access to an online discussion forum (32)	Waitlist (30)	Open access over 12 weeks with 6 modules (12 weeks)	Women with diagnosed breast cancer (mean time since diagnosis was 23.2±28.4 months for the treatment group and 31.8±37.6 months for the control group)	Active: 52.5±8.6 Control: 51.3±10.5	100	IES	Active: 1.10±0.6 Control: 0.96±0.6
Possemato et al. (2011)	Web-based expressive emotional writing with no guidance (15)	Web-based time management with no guidance (16)	3 sessions lasting 20 minutes (1 month post baseline and 3 months post baseline)	Veterans meeting DSM-IV criteria for PTSD or subthreshold PTSD; time post trauma not specified	34±11	81	PCL-M	Total sample: 46±14

Study: author (year)	Active Intervention (n)	Control (n)	Treatment duration (follow-up duration)	Population	Mean age (age range) in years	% female	Measure	Baseline scores
Possemato et al. (2010)	Web-based expressive emotional writing with no guidance (22)	Web-based factual writing with no guidance (26)	4 sessions: 3 intervention sessions lasting 15 minutes each and 1 assessment session, over 10 day period (3 months)	Kidney transplant recipients (>18 years)	46±12 (20-70)	54	PCL-C	Active: 37±12<br>Control: 37±1
Sayer et al. (2015)	Web-based non-TF expressive writing with no guidance (508)	C1: Web-based factual writing with no guidance (507)<br><br>C2: TAU (277)	4 20 minute sessions over 10 days (3 months and 6 months)	Veterans self-reporting problems with readjusting back into civilian life; time post trauma not specified	Active: 36.9±10.1<br>C1: 37.11±9.9<br>C2: 36.5±9.1	39.3	PCL-M	Active: 39.1±16.5<br>C1: 38.8±16.2<br>C2: 40.4±15.5
Schoorl et al. (2013)	Web-based attention bias modification training with no guidance (48)	Web-based attention training with no guidance (54)	8 sessions each lasting 15 minutes over 3 weeks (3 weeks)	Men and women on a waiting list for a mental health service meeting DSM-IV criteria for PTSD; >3 months post trauma	Active: 36.8±11.4<br>Control: 37.3±11.7	75.5	CAPS<br><br>SRIP	Active: 80.7±16.4<br>Control: 80.5±18.7<br><br>Active: 62.1±9.6<br>Control: 63.9±8.3
Spence et al. (2011)	Web-based TF-CBT with an online discussion forum and immediate (via instant messenger) and delayed (via discussion forum) tailored feedback (23)	Waitlist (21)	7 sessions, one per week (8 weeks)	Community-based sample meeting DSM-IV diagnosis of PTSD; >3 months post trauma	42.6±13.1 (19-65)	81	PCL-C	Active: 60.8±10.0<br>Control: 57.0±9.7
Spence et al. (2014)	Web-based TF-CBT with tailored feedback (59)	Web-based non-TF-CBT with delayed therapist feedback (66)	TF-CBT: 6 sessions<br>Non-TF-CBT: 4 sessions, each lasting 10-20 minutes, one per week (9 weeks post baseline, 3 months follow-up)	Community based sample scoring high on a measure of PTSD; >1 month post trauma	41±11.4 (21-68)	86	PSS<br><br>IES-R	Active: 32.0±9.3<br>Control: 31.7±9.6<br><br>Active: 51.3±15.7<br>Control: 46.2±14.7<br>TF-
Steinmetz et al. (2012)	Web-based non-TF-CBT with no guidance (18)	C1: Web-based psychoeducation with no guidance (19)<br><br>C2: TAU including standard physical and mental healthcare provision (19)	Open access to website over a 1 month period, 6 modules (1 month)	Men and women with experience of a specific natural disaster 11 months prior to the study who were scoring high on a measure of PTSD	Active: 43.1±11.5<br>C1: 40.5±11.1<br>C2: 45.4±10.7	85.7	MPSS	Active: 26.7±17.4<br>C1: 29.8±18.4<br>C2: 28.3±18.4
Wagner et al. (2006)	Web-based TF-CBT with tailored feedback (26)	Waitlist (25)	2 sessions per week lasting 45 minutes, over 5 weeks; 10 sessions in total (6 weeks)	Men and women who had experienced a death of a significant other (mean time post bereavement was 4.6±6.6 years) and scored high on a measure of PTSD	37.0±10.2 (19-68)	92.7	IES	Intrusions<br>Active: 24.3±6.8<br>Control: 26.6±4.9<br><br>Avoidance:<br>Active: 17.2±16.7<br>Control: 16.7±9.7

Study: author (year)	Active Intervention (n)	Control (n)	Treatment duration (follow-up duration)	Population	Mean age (age range) in years	% female	Measure	Baseline scores
Wang et al. (2013)	Web-based Non-TF-CBT<br>T1: Urban/No guidance (46)<br>T2: Rural/technical support and automated electronic messages (49)	Waitlist (44 urban, 44 rural)	Open access to website over a 1 month period, 6 modules (1 month)	Community based sample meeting DSM-IV criteria for PTSD; >3 months post trauma	18-70	72.6	PDS	T1: 1.7±0.6<br>Control: 1.7±0.6<br><br>T2: 1.8±0.5<br>Control: 1.8±0.6
Winzelberg et al. (2003)	Web-based semi-structured moderated online discussion forum (36)	Waitlist (36)	12 weeks of open access with new topic for discussion presented each week, 3 consecutive groups (12 weeks)	Women diagnosed with primary breast carcinoma diagnosis (≤32 months, mean=12±9)	49.5±6.2 (30-69)	100	PCL-C<br><br>PSS	Active: 32.5±10.0<br>Control: 33.1±12.1<br><br>Active: 16.4±7.3<br>Control: 16.0±7.4
Zernicke et al. (2014)	Web-based mindfulness-based cancer recovery intervention with immediate tailored feedback (30)	Waitlist (32)	8 weekly 2-hour sessions with opportunity to access resources in between, e.g. daily 45 minute mediation/yoga exercises encouraged (8 weeks)	Men and women ≤3 years since completed primary cancer treatment scoring high on a measure of psychological distress	Active: 58±8.2, Control: 58±13.0 (29-79)	73	CSOSI	Active: 59.7±32.5<br>Control: 66.1±33.8

C1=control 1; C2=control 2; CAPS=clinician-administered PTSD scale; CSOSI=Calgary symptoms of stress inventory; DSM-IV=diagnostic statistical manual 4th edition; IES=impact of events scale; IES-D=impact of events scale Dutch version; IES-R=impact of events scale-revised; MI=motivational interviewing; MPSS=modified PTSD symptom scale; Non-TF-CBT=non trauma focussed cognitive behavioural therapy; PCL-5=PTSD checklist-5; PCL-C=PTSD checklist civilian version; PCL-M=PTSD checklist military version; PDS=posttraumatic diagnostic scale; PSS=posttraumatic stress scale; PTSD=post-traumatic stress disorder; SRIP=self-rating inventory for posttraumatic stress disorder; STSS=secondary traumatic stress scale; T1=treatment 1; T2=treatment 2; T3=treatment 3; TAU=treatment as usual; TES=traumatic event scale; TF-CBT=trauma focussed cognitive behavioural therapy.
